# Copy number variation in the region harboring *SOX9* gene in dogs with testicular/ovotesticular disorder of sex development (78,XX; *SRY*-negative)

**DOI:** 10.1038/srep14696

**Published:** 2015-10-01

**Authors:** Malgorzata Marcinkowska-Swojak, Izabela Szczerbal, Hubert Pausch, Joanna Nowacka-Woszuk, Krzysztof Flisikowski, Stanislaw Dzimira, Wojciech Nizanski, Rita Payan-Carreira, Ruedi Fries, Piotr Kozlowski, Marek Switonski

**Affiliations:** 1 European Centre of Bioinformatics and Genomics, Institute of Bioorganic Chemistry, Polish Academy of Sciences, Poznan, Poland; 2 Department of Genetics and Animal Breeding, Poznan University of Life Sciences, Poznan, Poland; 3 Chair of Animal Breeding, Technische Universität München, Germany; 4 Chair of Livestock Biotechnology, Technische Universität München, Germany; 5 Department of Pathology, Wroclaw University of Environmental and Life Sciences, Wroclaw, Poland; 6 Department of Reproduction and Clinic of Farm Animals, Wroclaw University of Environmental and Life Sciences, Wroclaw, Poland; 7 CECAV, Centro de Ciência Animal e Veterinária, Universidade de Trás-os-Montes e Alto Douro, Quinta de Prados, Vila Real, Portugal

## Abstract

Although the disorder of sex development in dogs with female karyotype (XX DSD) is quite common, its molecular basis is still unclear. Among mutations underlying XX DSD in mammals are duplication of a long sequence upstream of the *SOX9* gene (RevSex) and duplication of the *SOX9* gene (also observed in dogs). We performed a comparative analysis of 16 XX DSD and 30 control female dogs, using FISH and MLPA approaches. Our study was focused on a region harboring *SOX9* and a region orthologous to the human RevSex (CanRevSex), which was located by *in silico* analysis downstream of *SOX9*. Two highly polymorphic copy number variable regions (CNVRs): CNVR1 upstream of *SOX9* and CNVR2 encompassing CanRevSex were identified. Although none of the detected copy number variants were specific to either affected or control animals, we observed that the average number of copies in CNVR1 was higher in XX DSD. No copy variation of *SOX9* was observed. Our extensive studies have excluded duplication of *SOX9* as the common cause of XX DSD in analyzed samples. However, it remains possible that the causative mutation is hidden in highly polymorphic CNVR1.

The most common canine disorder of sex development (DSD) manifests as testes or ovotestes without gametogenic activity, normal female karyotype (78,XX) and lack of the *SRY* gene^1^. This abnormality, termed testicular or ovotesticular XX DSD, is also quite common in other mammals, including humans^2^ and livestock species – goat, pig, horse^3^.

The genetic basis of XX DSD phenotype is not uniform between mammalian species. In humans heterozygous duplication and triplication of a long sequence (approx. 78 kb) located 0.5 Mb upstream of *SOX9*, called RevSex^4^,^5^,^6^ (in this study called HumRevSex) or testis specific *SOX9* enhancer candidate region^7^ are considered as the causative mutations. Recently the XX DSD HumRevSex region was delimited to 68 kb and distinguished from the XY DSD RevSex region^8^. This region likely contains an enhancer regulatory sequence which duplicated may trigger *SOX9* expression in the absence of the *SRY* gene product^9^. In a single case of testicular XX DSD in roe deer three copies of the entire *SOX9* gene, including 5′- and 3′-UTR, were detected by quantitative PCR (qPCR)^10^. In pigs, genome wide association study (GWAS) of related animals with XX DSD implicated the *SOX9* region, but did not pinpoint the causative mutation^11^.

Other regions have also been implicated in XX DSD. The first causative mutations were identified in human *RSPO1* gene, which is involved in ovarian development in humans^12^. In goats a 11.7 kb deletion near another gene (*FOXL2*), also important for ovarian development, is responsible for this disorder^13^,^14^. Finally, the duplication of *SOX3* gene has been identified in humans with XX DSD phenotype^15^.

There have been numerous attempts to identify the causative mutation or linked genetic markers in dogs, but so far without success [reviewed by^1^,^16^,^17^]. It has recently been claimed that some canine XX DSD cases are caused by duplication of a 577 kb region containing the *SOX9* gene, as detected by array comparative genome hybridization (aCGH) and confirmed by real time qPCR. The mutation was detected in two of seven XX DSD dogs analyzed and study of control samples was not performed^18^. Although, it has to be noted that duplication of *SOX9* in control samples was not detected in any of copy number variants (CNVs) discovery studies^19^,^20^,^21^,^22^,^23^.

Dogs show exceptional phenotypic variability and a high frequency of hereditary diseases, some of which are known to be due to structural variations in the genome^24^. Identification of copy number variable regions (CNVRs) in the dog is thus of particular interest. Rossi *et al.*^18^ suggested a potential association between XX DSD and a duplication in the *SOX9* region, and we considered this worthy of further investigation. Here we describe the use of cytogenetic mapping (fluorescence *in situ* hybridization, FISH) and multiplex ligation-dependent probe amplification (MLPA) approaches to identify two highly variable CNVRs, and show that *SOX9* is not duplicated in any of the samples analyzed.

## Results

### *In silico* analysis of the *SOX9* region

**Figure 1:**
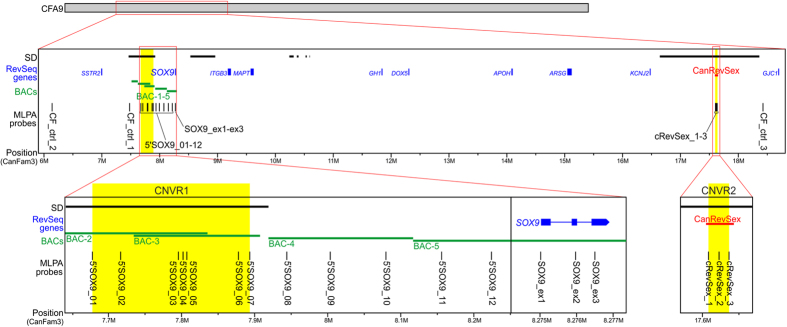
Map of the region studied. From top to bottom are indicated: segmental duplication (SD) according to Nicholas *et al.*^20^, RevSeq genes (blue), FISH probes (green) and MLPA probes (black) with IDs indicated next to or below. The *SOX9* with the upstream region and CanRevSex region are shown at higher resolution. Regions highlighted in yellow correspond to CNVRs detected in this study. The position of selected elements is indicated under the panels (please note that the scale is different for each panel).

Since it is known that critical regulatory region for human *SOX9* is located approx. 0.5 Mb upstream of the gene we anticipated a similar location for regulatory region in the dog genome. Unexpectedly, sequence similarity analysis of HumRevSex and canine reference genome (http://genome.ucsc.edu/cgi-bin/hgBlat, BLAT, UCSC GB) revealed that the predicted position of the canine sequence, termed CanRevSex (chr9:17605057-17642567, CanFam3.1), is very different, being located more than 9 Mb downstream of *SOX9* (Fig. 1). CanRevSex also spans only about 37 kb (part of HumRevSex) and shows relatively low homology to HumRevSex (86.1%). CanRevSex and HumRevSex differ in their sequence and genomic location, but it is not clear whether they are similar in function. We thus decided to analyze the region upstream of *SOX9* and the downstream CanRevSex region, using FISH and MLPA for locus-specific CNVs analysis.

### Analysis of the *SOX9* region by FISH

Cytogenetic study of the *SOX9* upstream region commenced with verification of BAC clones on one animal with XX DSD and one healthy reference female dog (data not shown). The BAC-5 clone, located closest to *SOX9*, produced specific and typical hybridization signals on CFA9. The second clone (BAC-4) in the contig order, upstream of the *SOX9* gene (Fig. 1), also produced typical hybridization signals on CFA9, but additional signals were found on a long arm of the chromosome X (CFAXq), where there is a block of constitutive heterochromatin. The BAC-3 clone showed variation of the hybridization signals. A typical signal was present on CFA9 in a reference female dog, while in the XX DSD case the signal appeared in only one copy of the CFA9. Signals were also detected on one small autosome, which was identified as CFA18 by *in silico* analysis of sequence homology. Signals on CFA18 were observed in XX DSD and reference dogs. Another two clones (BAC-1 and BAC-2) showed hybridization signal at the same position as the BAC-4 clone. BAC-1 and BAC-2 signals were observed at the expected position on CFA9, and additional signals on the long arm of the X chromosome. The BAC-2 signal intensity showed variability, but because the probe bound to other chromosomes, it was not deemed a reliable indicator for CNV analysis.

**Figure 2:**
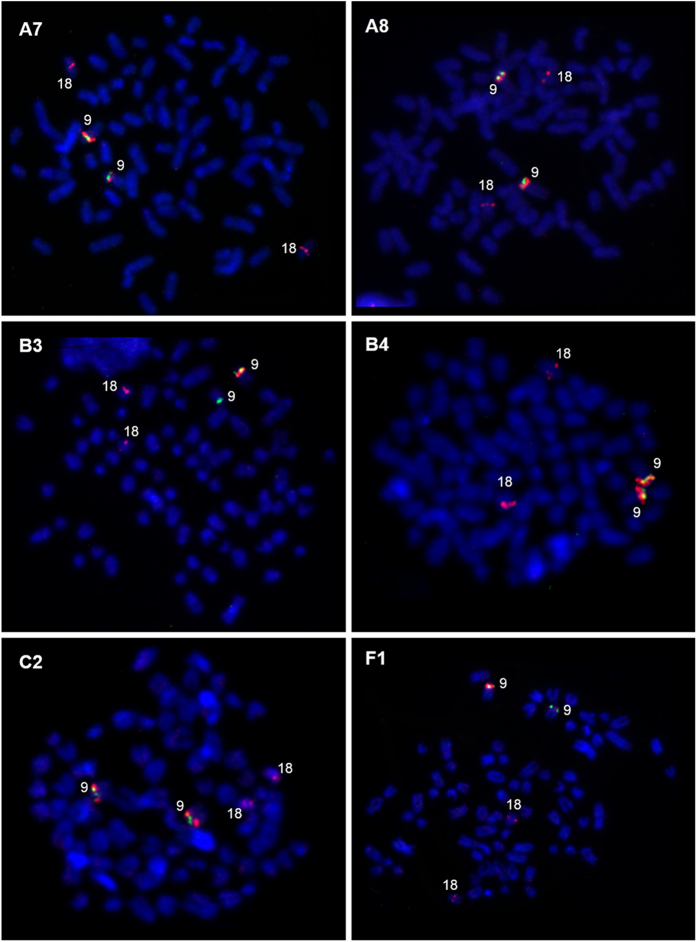
Representative images of FISH analysis with two BAC clones: BAC-5 (green) and BAC-3 (red), for 6 animals. Red signals were observed on CFA9 and CFA18, while the green signal was present only on CFA9. Size variation of the red FISH signal in CFA9 was described as: large (++), normal (+) and lack of the signal (−). The following FISH patterns are visible: A7 (++/+), A8 (++/++), B3 (++/−), B4 (++/++), C2 (++/++) and F1 (++/+).

Results of the FISH experiment indicated a potential CNVR in the region detected by BAC-3. To investigate this further, four groups of animals were included in the FISH study: XX DSD dogs (A5, A6, A7, A8, B3, B4), healthy relatives of A6 and B4 XX DSD dogs (C2, C3, C4 and C5) and 4 reference, fertile female dogs (F1–F4). Dual-colour FISH was applied, using two probes, BAC-3 and BAC-5. The BAC-3 clone was used as a probe for CNV detection (labelled in red), while BAC-5 was used as a CFA9-specific reference probe (labelled in green). The hybridization signal produced by the BAC-3 probe showed variation in the animals studied (Fig. 2). Two large signals on CFA9 were found in A5, A6, A8, B4 and C2 animals, a large signal on one copy of CFA9 and a weak signal on the second copy of CFA9 were observed in A7, C3, C4 and C5, while in B3 the signal was visible on only one copy of CFA9. Similar variation was observed on CFA9 in reference animals (F1–F4). These results suggested that hybridization signal variability is a common variation in this species. Inheritance of the CNV was confirmed in Pug family.

### Analysis of the *SOX9* and CanRevSex regions by MLPA

To investigate the canine *SOX9* region in more detail, we developed a custom-made CanSOX9+ MLPA assay composed of 21 probes and covering the following sub-regions of interest: i) *SOX9* gene, ii) the region of ~620 kb in length upstream of the *SOX9* gene and iii) the 37,5 kb CanRevSex region (Fig. 1).

**Figure 3:**
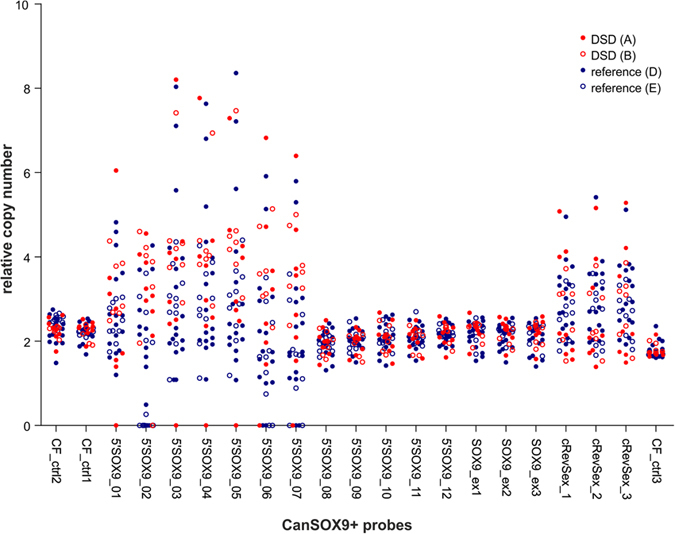
MLPA results for all tested samples. The column scatter plot represents the relative copy number (y-axis) of each CanSOX9+ probe (x-axis). Each dot represents one sample. Colored dots represent samples of XX, SRY-negative, testicular or ovotesticular DSD females (group A, solid red dots) and XX, SRY-negative DSD females with unknown gonad histology (group B, open red dots). Blue dots represent reference females (groups D and E).

MLPA analysis was performed on 45 dog samples from 21 different breeds (for details see Table 1). Results for individual samples are shown as bar plots in Suppl. Fig. S1 and summarized in the column scatter plot in Fig. 3. As shown in Fig. 3, three probes located in the CanRevSex region (cRevSex_1-cRevSex_3) and seven probes located in the region 0.4 Mb upstream of *SOX9* (5′SOX9_01-5′SOX9_07) show very high signal variation (average coefficient of variation (aCV) = 0.55, compared to average CV = 0.14 and CV = 0.11 in non-polymorphic and control regions, respectively), indicating the extensive copy number (CN) variation, including homozygous deletions, in these regions. This result shows that two CNVRs are present in the studied SOX9 region: CNVR1 upstream of *SOX9* gene of a minimal size of 214 433 bp, and CNVR2 in the CanRevSex region of a minimal size of 27 961 bp. The relative CN of these regions ranges from 0 to over 8 copies. Also, as shown in Fig. 3 and S1, there is no sign of CNVs within the *SOX9* gene (average CV = 0.14). To confirm this result, we replicated our analysis with the modified CanSOX9+ assay excluding probes located in detected CNVRs. As Suppl. Fig. S2 shows, this confirmed the absence of CNV in *SOX9* gene in the analyzed samples.

As shown in Suppl. Fig. S1 CNVs do not occur at random in the variable regions, but the identified CNVs often extend over several consecutive probes. Among the most common CNVs observed are: i) gains of the region from probe 5′SOX9_03 to probe 5′SOX9_07 (5′SOX9_03-07); ii) deletions of probes 5′SOX9_02 and 5′SOX9_06-07; or iii) gains of CanRevSex region (cRevSex_1-3). Although the pattern of the observed variants is relatively conservative, it has to be noted that the determined CN values are relative and may differ somewhat depending on the reference samples used.

Detailed analysis of the variation observed in the regions tested showed that extensive CN variation occurs both in affected and control samples and that there is no unambiguous relationship between observed CNVs and the XX DSD phenotype (Fig. 3). Although we observed some specific CNV patterns for different breeds, especially for the CanRevSex region (Suppl. Fig. S3), most CNVs occurred in more than one breed and were not breed-specific.

Due to the lack of a simple relationship between CNVs and XX DSD phenotype, we decided to compare the relative signal distribution between samples from two groups of animals: XX DSD females (group A + B) and healthy females (group D + E) (Suppl. Fig. S4). This analysis demonstrated that in the region covered by six consecutive probes (5′SOX9_02-07) XX DSD animals showed increased average CN values compared to control samples. The strongest association (the highest signal difference between groups) was observed for probe 5′SOX9_02, which showed a significantly higher average relative signal in XX DSD females than in healthy females (3.23 vs 1.54, respectively; Mann-Whitney U test, p value = 0.0008). Similar CN differences were observed when affected animals (group A + B) were compared only with animals from group D, and no substantial differences were observed between groups D and E. These results suggest that the causative mutation for XX DSD may be located in this region. It has to be noted, however, that the observed association may result from heterogeneity and different breed composition of compared groups.

**Figure 4:**
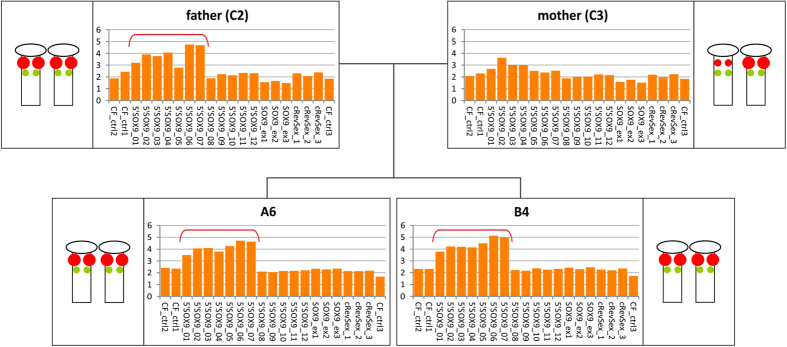
The MLPA and FISH analysis in the Pug family. The family consisted of two XX, SRY-negative DSD females (A6 and B4) and their healthy parents: father (C2) and mother (C3). Bar plots represent the relative copy number (y-axis) of each CanSOX9+ probe (x-axis). The red bracket marks probes, where the signal is increased (5′SOX9_01–5′SOX9_07). Corresponding diagrams of the results of FISH analysis are shown next to the MLPA bar plots.

We then analyzed the segregation of copy number changes in Pug family, which consisted of two affected siblings and their healthy parents (Fig. 4). We did not analyze grandparents of the affected siblings, due to the lack of grandfather DNA (C5) and the repeatedly poor quality of MLPA (non interpretable) results of the grandmother sample (C4). Both XX DSD animals (A6 and B4) showed a clear signal increase for probes 5′SOX9_01-07. A similar signal increase was observed in the father (C2), but not the mother (C3). The increased MLPA signal correlated with results of the FISH analysis. Unfortunately, we could not analyze other littermates, and were thus unable to conclude, whether the increase of copies in XX DSD animals represents an association or is coincidental.

## Discussion

Mutations in the region upstream of *SOX9* are responsible for several human genetic diseases^25^, including XX DSD, which is associated with duplications or triplications of the HumRevSex region located approx. 0.5 Mb upstream of *SOX9*^5^,^6^. Different techniques can be used to detect CNVRs^26^,^27^ and we applied two of them (FISH and MLPA) to compare region harboring *SOX9* in XX DSD dogs and normal, fertile female dogs.

FISH analyses revealed differences of the hybridization signal size for one of five BAC probes tested for 5′-flanking region of the dog *SOX9* gene. To visualize or confirm variation in this CNVR different types of FISH approaches can be applied using BAC or fosmid probes to detect FISH signal intensity^28^,^29^,^30^,^31^. We used a standard FISH procedure with BAC clones to find out whether different signal intensity variants exist among the XX DSD and control dogs. The size of inserts in the used BAC clones varied from 165–203 kb. The clone which revealed a very polymorphic fragment upstream of the *SOX9* (BAC-3) carried the 165 kb long insert. Due to a visible variation of the FISH signal we assumed that there is an extensive polymorphism in the region studied. An advantage of the FISH approach is visualization of CNVs size differences in homologous chromosomes, however, it does not indicate the precise CN in a locus of interest.

To precisely characterize CNVR detected by FISH, we employed the MLPA technique with a set of 21 probes designed to study the *SOX9* region (~650 kb) and the CanRevSex region (~40 kb), located ~9 Mb downstream of *SOX9*. This approach allowed us to conclude that there was no *SOX9* duplication in any of the samples tested. This observation contrasts with results of Rossi *et al.*^18^ who claimed that duplication of a large region, including entire *SOX9* gene, is a relevant cause of XX DSD. A similar association between *SOX9* duplication and XX DSD has also been reported for a roe deer^10^ and a boy^32^. However, both were single cases analyzed by qPCR. The lack of *SOX9* duplication in our relatively large cohort of XX DSD females at least suggests it is not a common cause of XX DSD in dogs.

The MLPA approach also allowed us to identify two highly variable regions: CNVR1 upstream of *SOX9*, and CNVR2 in the CanRevSex region downstream of *SOX9*. The location and observed variation of CNVR1 correlate well with the FISH analysis (BAC-3 probe). Because CNVs in both CNVRs were observed in affected and healthy animals they do not provide a simple explanation of the disorder. However, the association of the higher CN in the CNVR1 (from probes 5′SOX9_01 to 5′SOX9_07) with XX DSD phenotype suggests that this region may harbor CNV or some other genetic variation associated with XX DSD. This association is in line with our FISH results (BAC-3 probe) and with results of Pug family analysis in which high CN value in CNVR1 are observed in two affected siblings but not in the normal mother. The detection of two CNVRs in the *SOX9* region generally confirms previous reports. CNVR1 has been described previously in two genome-wide studies^20^,^33^ and CNVR2 in six genome-wide studies^19^,^20^,^22^,^23^,^33^,^34^ (Suppl. Fig. S5). Both CNVRs detected in this study co-localize with previously identified segmental duplications (SDs)^20^, which suggests they may have been formed by non-allelic homologous recombination (NAHR).

CNVR1 is located ~0.4–0.6 Mb upstream of *SOX9* and its approximate length is greater than 214 kb. It well overlaps with the size and the location of HumRevSex, which is located more than 500 kb upstream of *SOX9* and the length of this region is at least 67 kb^6^. However the *in silico* predicted position of CanRevSex, based on homology analysis, is ~9 Mb downstream of *SOX9*. This may suggest that the region critical for XX DSD is characterized not by sequence, but by its location. Interestingly, a very recent report of Rossi *et al.*^35^, based on FISH study and *in silico* analysis, suggested that canine genome assembly (canFam3) in the region of *SOX9* is not correct and the CanRevSex is in fact localized upstream of *SOX9*.

Our findings are consistent with previous studies performed on mouse models. The regulatory role of the upstream region (approx. 1 Mb) of *Sox9* was revealed in transgenic mouse, called the *Odds sex* mouse, which also carries an ~150 kb deletion upstream of *Sox9*. This modification triggered upregulation of *Sox9* in fetal gonads and XX mice, which developed as sterile males^36^. Further studies showed that promoter of the transgene, not the deletion itself, was responsible for the upregulation in XX *Odds sex* mice^37^. Extensive study on murine *Sox9* gene regulation revealed that its expression is crucial for Sertoli cell differentiation and is controlled by positive and negative regulators interacting with a 3.3 kb testis-specific enhancer (TES) that contains a highly conserved 1.4 kb sequence. The main positive regulators are SF1 and SRY transcription factors, and among crucial regulators are DAX1, WNT4, FOXL2, RESPO1 and β-catenin^38^.

Analysis of families in which the disorder segregates is a crucial step towards identifying its mode of inheritance and molecular characterization of the causative mutation. Extensive studies performed in goats revealed autosomal recessive inheritance^13^. Similar approach was applied to show the causative role of the *RSPO1* gene mutations in some cases of human XX DSD^12^. In pigs autosomal recessive inheritance was also proposed^39^ and candidate region containing the *SOX9* gene was indicated^11^. Interestingly, in XX DSD pigs no CNV was found in the 3.14 Mb region containing the *SOX9* gene. In humans pedigree analysis, associated with a causative 178 kb duplication located 600 kb upstream of *SOX9*, revealed autosomal dominant sex-limited (females only) inheritance. The inheritance mode of canine XX DSD is unclear. Originally, it was hypothesized that this disorder is inherited according to an autosomal recessive, sex-limited model^40^. The deleterious effect of *SOX9* duplication suggested by Rossi *et al.*^18^ does not fit this model and indicated that also dominant, sex-limited mode of inheritance is possible, as it was earlier demonstrated in humans^18^. In our study we could only analyze parents of two full sibling XX DSD cases. Since other littermates were unavailable we cannot draw any conclusion concerning the inheritance model.

Concluding, in our study on the molecular background of XX DSD we analyzed a relatively large cohort of cases and controls. We did not observe CN variation of the *SOX9* gene, however we found two highly polymorphic CNVRs located upstream (CNVR1) and downstream (CNVR2) of *SOX9*. Although higher copy number in CNVR1 may be associated with XX DSD phenotype, it was detected in both cases and controls, and therefore cannot be considered as a causative mutation. We hypothesize that within CNVR1 may occur mutation site/sites responsible for ablation of binding sites for repressor or enhancer of the *SOX9*.

## Material and Methods

### Ethics statement

Tissue sampling and clinical studies were carried out according to standard Polish veterinary protocols. All animal experiments were approved by a local Bioethical Commission for Animal Care and Use in Poznan (Poland).

### Material

**Figure 5:**
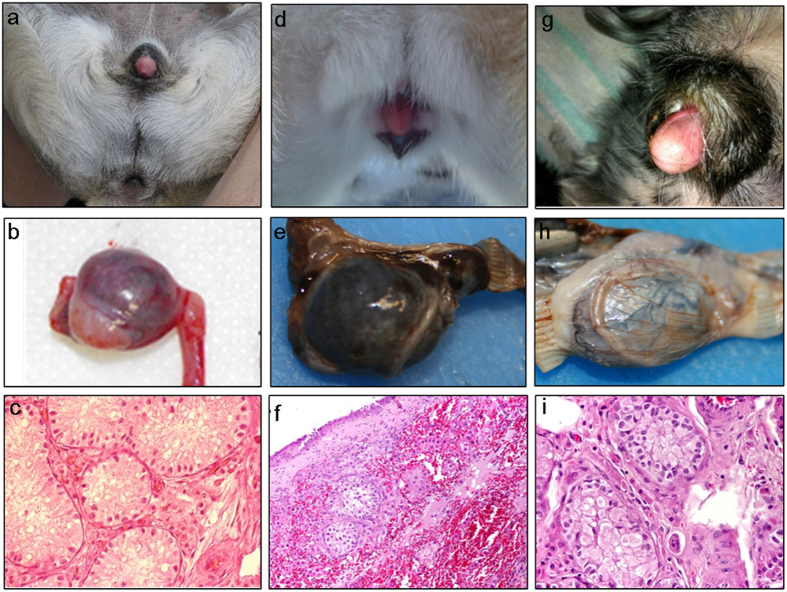
Characteristics of three new testicular (A6 and A8) or ovotesticular (A7) XX DSD cases. The following breeds were represented: Pug – case A6 (**a**–**c**), Beagle – case A7 (**d**–**f**) and Cocker Spaniel – case A8 (**g**–**i**). External genitalia (**a**,**d**,**g**), gonad morphology (**b**,**e**,**h**) and histology (**c**,**f**,**i**) are shown.

A total of 50 dogs were included in the study. These were grouped as follows: (A) 8 cases of unrelated XX DSD phenotypic females with enlarged clitoris and testis or ovotestis, this group was crucial in our study due to the knowledge of the gonads histology, (B) 8 cases of unrelated XX DSD phenotypic females with enlarged clitoris, but unknown gonad histology; (C) 4 ancestors of two testicular XX DSD full sibs, (D) 16 control females of the same breed as the XX DSD cases studied, (E) 10 control females of breeds where XX DSD has not yet been reported, and (F) 4 control females for the FISH study. Some XX DSD dogs were described earlier in terms of clinical examination, chromosome complement, presence of the *SRY* gene and histology of gonads, but were not studied by FISH and MLPA techniques (Table 1). Four new XX DSD cases, included in groups A (3) or B (1), were described for the first time (Fig. 5).

### Fluorescence *in Situ* Hybridization (FISH)

Chromosome preparations were obtained from short-term lymphocyte culture by standard procedures^41^. BAC clones covering the 5′ region of *SOX9* gene on canine chromosome 9 (CFA9) were selected based on localization upstream of *SOX9*, in a region which position corresponds to HumRevSex. The BACs were selected from CHORI-82 Canine Boxer (F) (Canis familiaris) BAC library (https://bacpac.chori.org/) with the use of UCSC Genome Browser (http://www.genome.ucsc.edu/). The following BAC clones were used in FISH experiments: BAC-1 (CH82-405G24), BAC-2 (CH82-496F06), BAC-3 (CH82-26L13), BAC-4 (CH82-135B15) and BAC-5 (CH82-116C01). The locations of the BAC probes are shown in Fig. 1.

DNA from BAC clones was isolated by alkaline lysis and labelled by random priming with biotin-16-dUTP or digoxigenin-11-dUTP. FISH hybridization was performed according to Szczerbal *et al.*^42^. Briefly, denatured probes were applied to a denatured chromosome preparation and hybridized overnight at 37 °C. Signal detection and amplification were performed using streptavidin-Cy3 or anti-digoxigenin-fluorescein. Chromosomes were counterstained with 4′,6-diamidino-2-phenylindole (DAPI) and the standard chromosome nomenclature of the dog^43^ was applied for localization of the probes on CFA9. Hybridization signal of BAC5 probe, specific to the region of interest, was used as a marker of CFA9.

Slides were analyzed under a Nikon E 600 Eclipse fluorescence microscope, equipped with a cooled digital CCD camera, driven by the computer-aided software LUCIA.

### Multiplex Ligation-dependent Probe Amplification (MLPA)

A custom-made MLPA assay (CanSOX9+ assay) was designed and generated for detailed analysis of CNVRs in the canine region harboring *SOX9*. The CanSOX9+ assay was composed of 21 probes (Fig. 1, Suppl. Table S1) including: i) three control probes (CF_ctrl1-CF_ctrl3) located on CFA9, outside the investigated region, in locations with no evidence of common CNVRs^19^,^20^,^22^,^23^,^33^,^34^ and 18 probes covering the investigated *SOX9* region, including: ii) three probes located in the *SOX9* gene, one probe in each exon (SOX9_ex1-SOX9_ex3); iii) 12 probes evenly distributed along the 620 kb-long region upstream of *SOX9* (5′SOX9_01-5′SOX9_12) (the less even distribution of probes in the fragment overlapped by BAC-3 is a consequence of segmentally duplicated sequences with high homology to sequences on CFA18); and iv) three probes located in the region homologous to HumRevSex region (CanRevSex) (cRevSex_1-cRevSex_3). The MLPA probes and general probe layout were designed according to the strategy previously proposed by Kozlowski *et al.*^44^ and by Marcinkowska *et al.*^45^, which involves short oligonucleotide probes easily generated via standard chemical synthesis. The total probe length ranged from 93 to 164 nt. Target sequences for all probes were designed according to the CanFam2 and CanFam3.1 dog genome assemblies and were selected to avoid SNPs, repeat elements and sequences of extremely high or low GC content. All MLPA probes were designed to be specific for the unique genomic sequence. In some cases (7 probes), in regions with high homology to other chromosomes, the MLPA probes were designed to specifically recognize only the region of interest on CFA9. Suppl. Table S1 shows detailed characteristics and sequences of the probes used. All MLPA probes were synthesized by Integrated DNA Technologies (Skokie, IL, USA).

MLPA reactions were run according to the manufacturer’s general recommendations (MRC-Holland, Amsterdam, Netherlands), as described previously in^46^ and^44^. Briefly, approximately 100 ng DNA was denatured and hybridized with MLPA probe-mix for 16 hours. In the next step, all properly hybridized probes were ligated and then amplified using the pair of universal primers. PCR products were separated via capillary electrophoresis on an ABI Prism 3130XL apparatus (Applied Biosystems, Carlsbad, CA, USA) and electropherograms analyzed using GeneMarker software (v1.91). The signal intensities (peak heights) of all probes were transferred to the prepared Excel spreadsheets in which further analyses were performed. To avoid run-to-run signal variation, signals of each probe in each sample were normalized by division by the average signal of control probes. The normalized MLPA signal of all samples was then compared to the normalized signal of samples selected as references. Reference samples were randomly selected from the group of healthy females (D) that showed the lowest normalized signal variation for all probes and no homozygous deletions for any of the regions investigated.

All MLPA reagents, except for the probe mix, were purchased from MRC-Holland (Amsterdam, the Netherlands). All graphs shown were generated using Prism v. 4.0 (GraphPad, San Diego, CA, USA) and Microsoft Excel.

## Additional Information

**How to cite this article**: Marcinkowska-Swojak, M. *et al.* Copy number variation in the region harboring SOX9 gene in dogs with testicular/ovotesticular disorder of sex development (78,XX; *SRY*-negative). *Sci. Rep.* **5**, 14696; doi: 10.1038/srep14696 (2015).

## Supplementary Material

Supplementary Information

## Figures and Tables

**Table 1:** Characterization of the dogs studied.

Animal	Breed	Reference	Technique applied	
			FISH	MLPA
**Group A** – testicular or ovotesticular 78,XX and *SRY*-negative DSD phenotypical females with enlarged clitoris				
A1	Cocker spaniel	47	−	+
A2	Bernese Mountain Dog	48	−	+
A3	American Staffordshire Terrier	16	−	+
A4	American Staffordshire Terrier	49	−	+
A5	Leonberger	16	+	+
A6	Pug	This study	+	+
A7	Beagle	This study	+	+
A8	Cocker spaniel	This study	+	+
**Group B** – 78,XX and *SRY*-negative DSD phenotypical females with enlarged clitoris but with unknown histology of gonads				
B1	American Staffordshire Terrier	16	−	+
B2	Tibetan Terrier	16	−	+
B3	French Bulldog	16	+	+
B4	Pug	This study	+	+
B5	American Staffordshire Terrier	49	−	+
B7	Miniature Pinscher	49	−	+
B8	German Shepherd	50	−	+
B9	Yorkshire Terrier	16	−	+
**Group C** – Ancestors of 2 fullsibs with DSD (A6 and B4)				
C2	Pug, father of A6 and B4	This study	+	+
C3	Pug, mother of A6 and B4	This study	+	+
C4	Pug, father of C2 and C3	This study	+	+
C5	Pug, mother of C2	This study	+	−
**Group D** - 16 control females representing breeds in which DSD has been reported: American Staffordshire Terrier (4), Beagle (2), German Shepherd, Cocker Spaniel (2), Pug (2), Bernese Mountain Dog, Yorkshire Terrier (3) and French Bulldog			−	+
**Group E** - 10 control females representing breeds in which DSD has not so far been reported: Rottweiler, Poodle, Mastiff, Pekingese, Bichon Friese, Chihuahua, Boxer, Dachshund, Briard, Labrador			−	+
**Group F** – 4 control females used for FISH analysis: Akita, Irish Setter, Shih Tzu and mongrel			+	−
