# Supplementary material for: Copy number variation in the region harboring SOX9 gene in dogs with testicular/ovotesticular disorder of sex development (78,XX; SRY-negative)
Source: Sci Rep. 2015 Oct 1;5:14696. doi: 10.1038/srep14696 (PMC4589768; doi:10.1038/srep14696)
Supplement: Supplementary Information [file srep14696-s1.doc]

SUPPLEMENTARY INFORMATION TO

**Copy number variation in the region harboring *SOX9* gene in dogs with testicular or ovotesticular disorder of sex development (78,XX; *SRY*-negative)**

Malgorzata Marcinkowska-Swojak^1†^, Izabela Szczerbal^2†^, Hubert Pausch^3^, Joanna Nowacka-Woszuk^2^, Krzysztof Flisikowski^4^, Stanislaw Dzimira ^5^, Wojciech Nizanski^6^, Rita Payan-Carreira^7^, Ruedi Fries^3^, Piotr Kozlowski^1*^and Marek Switonski ^2*^

**^†^** These authors contributed equally to this work

* Corresponding Authors


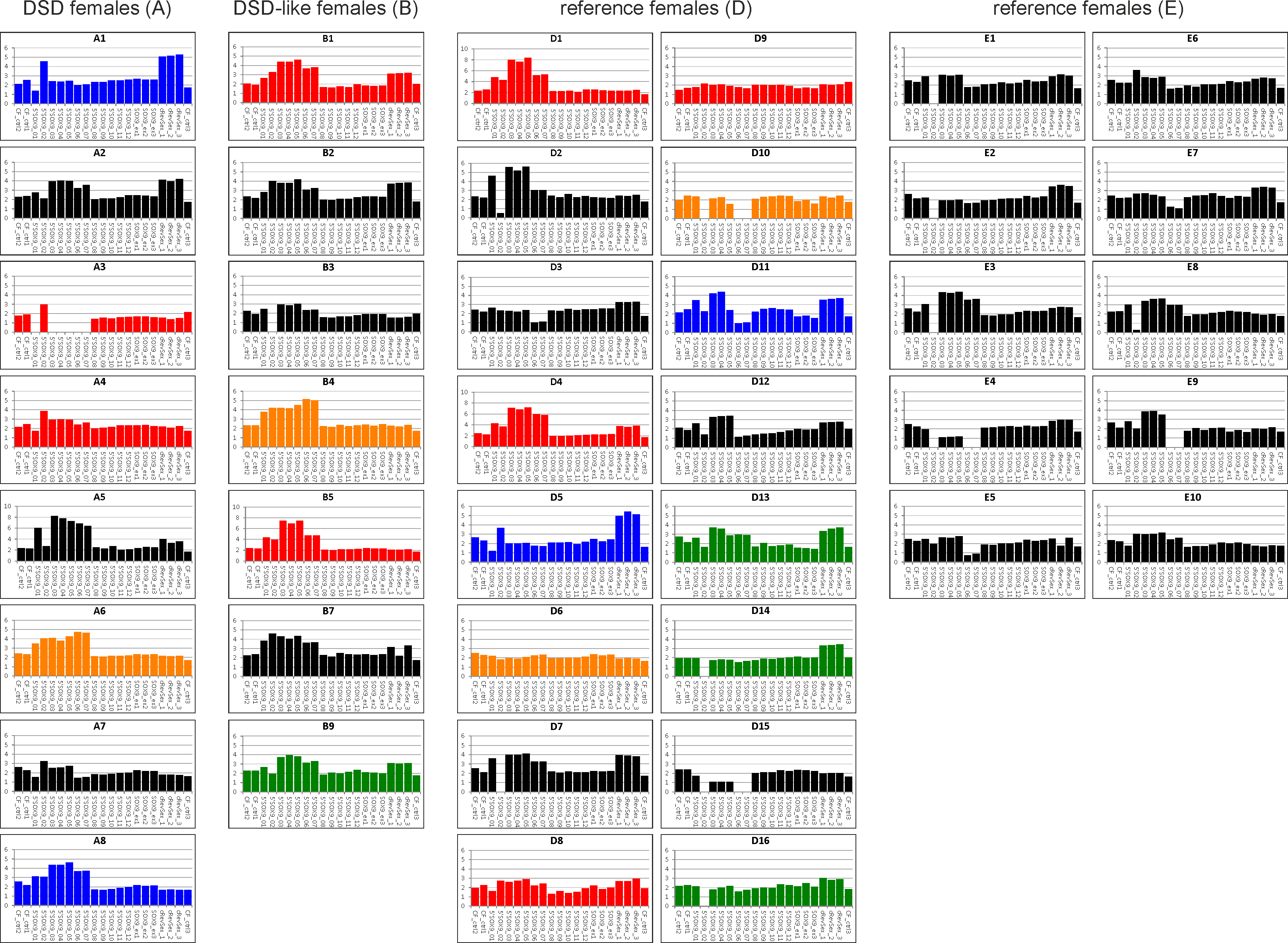


**Supplementary Figure S1.** MLPA results for individual tested samples. Bar plots represent the relative copy number (y-axis) of each CanSOX9+ probe (x-axis). The color of bar plot represents the breed from which samples were derived: American Staffordshire Terrier (red), Pug (orange), Cocker Spaniel (blue) and Yorkshire Terrier (green). Black bar plots represent animals derived from different breeds.


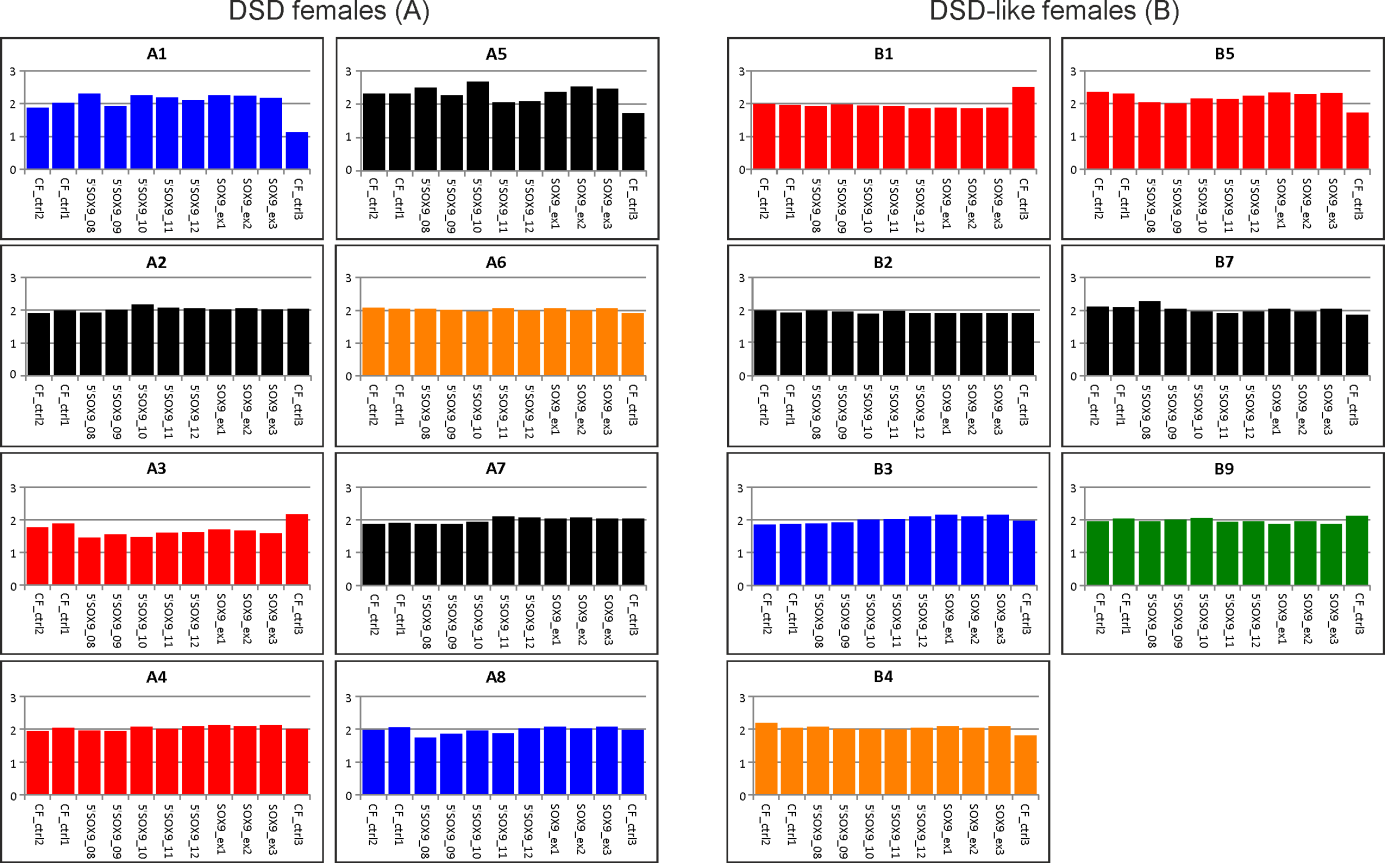


**Supplementary Figure S2.** MLPA analysis focused on the *SOX9* gene (excluding CNVRs). Bar graphs show the results of MLPA analysis with the minimized CanSOX9+ assay, excluding probes located in detected CNVRs in groups of XX DSD animals (A and B). Breeds are colored as in Supplementary Figure S1.


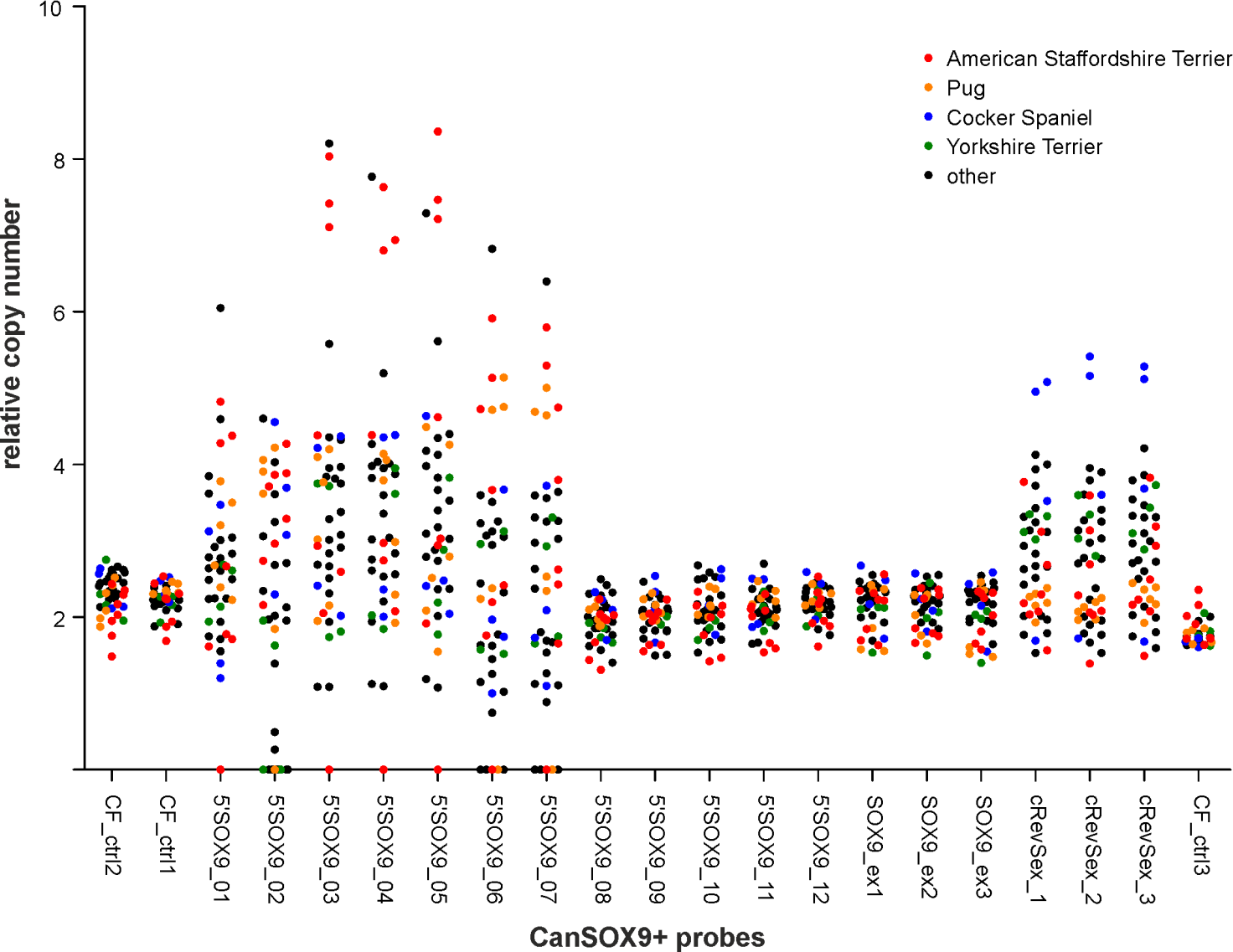


**Supplementary Figure S3.** MLPA results for samples divided by breed. The column scatter plot represents the relative copy number (y-axis) of each CanSOX9+ probe (x-axis). Each dot represents a sample. Breeds are colored as in Supplementary Figure S1.


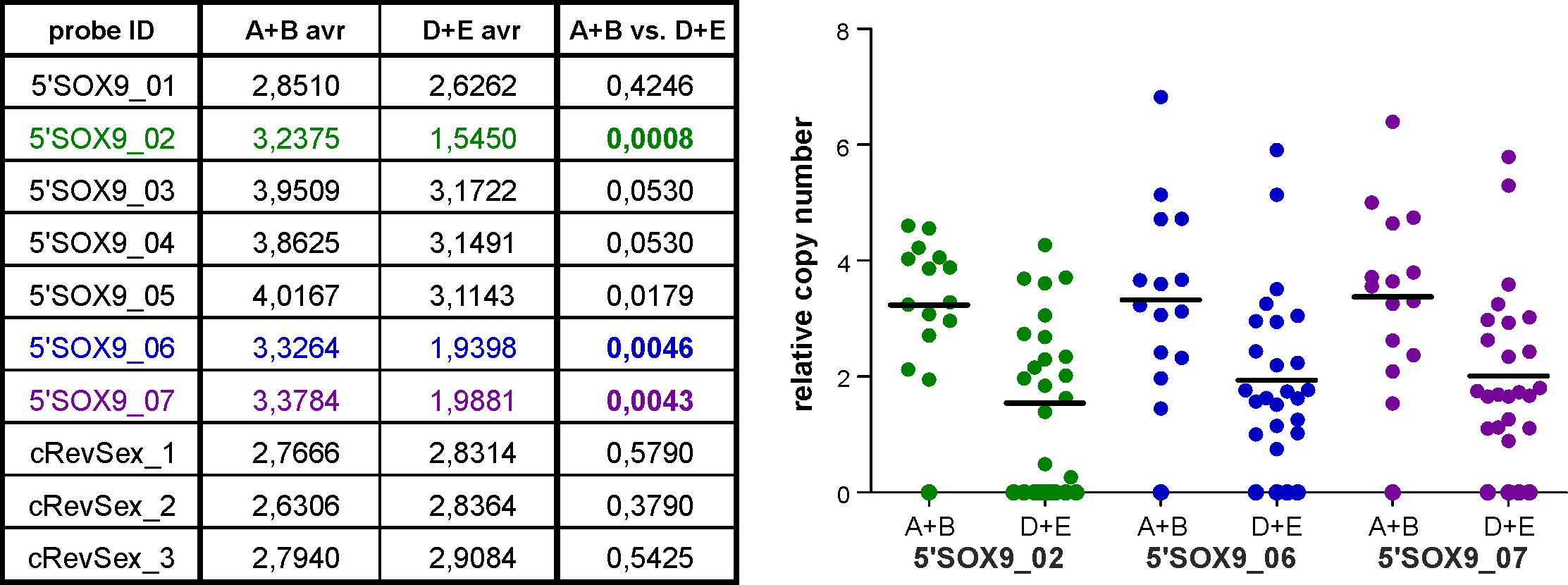


**Supplementary Figure S4.** Relative signal distribution in samples from group A+B and D+E. The table shows the average relative copy number of variable CanSOX9+ probes (5’SOX9_01-5’SOX9_07 and cRevSex_1-cRevSex_3) in: i) XX DSD females (group A+B) (second column) and ii) healthy control females (group D+E) (third column). The fourth column shows differences in average relative copy number between these groups. The most significant are marked in bold. The first column shows probe IDs. The column scatter plot next to the table shows the relative copy number (y-axis) of the three most variable CanSOX9+ probes (5’SOX9_02, 5’SOX9_06 and 5’SOX9_07) (x-axis) in the two groups of animals mentioned above. Each dot represents one sample. The black line in each group of dots indicates the mean value of the relative copy number in that group.


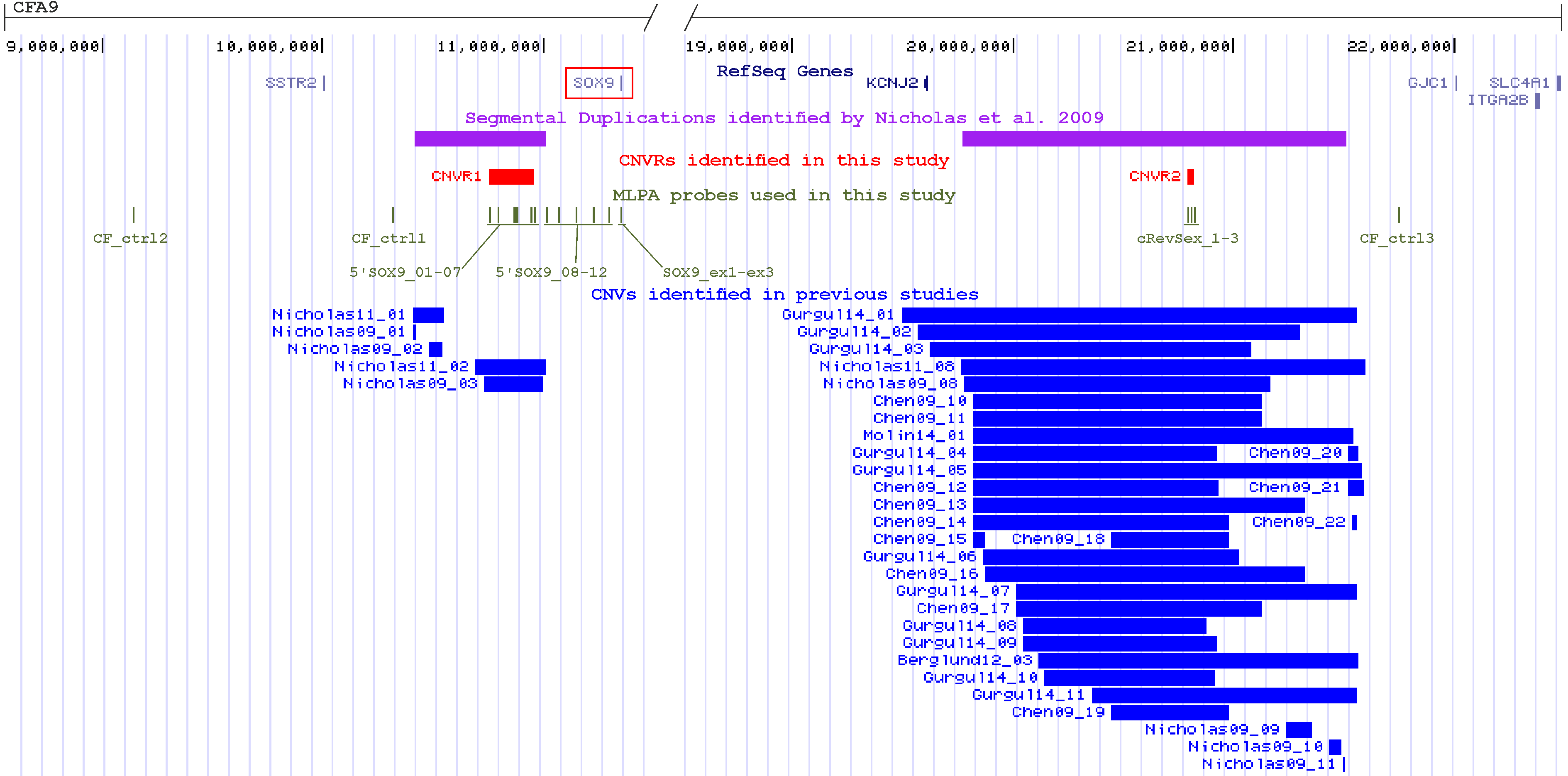


**Supplementary Figure S5.** Map of the structural variation identified on CFA9 in the SOX9 region (screenshot from UCSC GB with RefSeq UCSC track and annotated custom tracks). From the top are shown the following tracks: dark blue- RefSeq Genes (SOX9 gene was framed), purple- SDs identified by Nicholas et al. 2009^20^, red- CNVRs identified in this study: CNVR1 and CNVR2, green- CanSOX9+ probes used in this study, and blue- copy number variants (CNVs) identified in previous studies^19,20,22,23,33,34^. CNVs were number according to their occurrence on CFA9.

**Supplementary Table S1.** Detailed characteristics of CanSOX9+ MLPA assay.

**Legend:**

5'PSS, 3'PSS - 5' and 3' primer-specific sequence, respectively

5'SS, 3'SS - 5' and 3' stuffer sequence, respectively

5'TSS, 3'TSS - 5' and 3' target-specific sequence, respectively

Tm - melting temperature

5'HP - complete sequence of 5' half-probe

3'HP - complete sequence of 3' half-probe

5'HPL, 3'HPL - 5' and 3' half-probe length

SALSA PCR Forward primer (Labeled): *GGGTTCCCTAAGGGTTGGA

SALSA PCR Reverse primer (Unlabeled): GTGCCAGCAAGATCCAATCTAGA

Sequence used for generation of all 5' and 3' stuffer sequences: AC#V00604, Phage M13 genome, position 3-99

5'-cgctactactattagtagaattgatgccaccttttcagctcgcgccccaaatgaaaatatagctaaacaggttattgaccatttgcgaaatgtatctaatggtcaaactaaatctac-3'
